# Configural path of obesity: linkage of physical activity and lifestyle based on fuzzy-set qualitative comparative analysis

**DOI:** 10.3389/fpubh.2025.1533311

**Published:** 2025-03-28

**Authors:** Yi Wang, Fengshan Yue, Wei Li, Lixu Tang

**Affiliations:** ^1^School of Physical Education, Sichuan University, Chengdu, China; ^2^Department of Physical Education, Northwestern Polytechnical University, Xi’an, Shaanxi, China; ^3^School of Martial Arts, Wuhan Sports University, Wuhan, Hubei, China

**Keywords:** obesity, physical activity, lifestyle, qualitative comparative analysis of fuzzy sets, fsQCA

## Abstract

Obesity plays a significant role in the burden of various health conditions, it is not only a global health issue but challenges the national public health system. Some regions of China still face a high prevalence of obesity, and it is broadly recognized that physical activities interact with lifestyle in different pathways would affect obesity. We aim to capture different configurational paths that lead to obesity, using the fuzzy set Qualitative Comparative Analysis. Eight obesity-related variables were involved, and data were collected between January 1, 2021, and January 31, 2022. The study shows six configurational paths result in obesity, in which the necessary condition is “educational status,” and core conditions of “the time of exercise” and “weekly sitting time*sleeping time less than 6 h*second hand smoking exposure on average of 4–6 days per week *keep excising on average of 4 times per week* exercise intensity on the shortness of breath, markedly increased heart rate, heavy sweating” play an important role in the obesity outcome, and the solution exhibits acceptable consistency is 0.50. The six configurational paths solution consistency is 0.76, and the solution coverage is 0.31. Besides the necessary condition and core factors that play(s) an important role in the development of obesity, we have to consider the multiple factors of physical activity and lifestyle have a cross-cutting effect on obesity. This can offer a better understanding of the mechanisms that cause obesity by identifying and characterizing the regional population, which would help develop an effective protective measure for obesity.

## 1 Introduction

With the proposal and promotion of the construction of “Healthy China 2030,” which focuses on popularizing healthy living, optimizing health services, building healthy environments and developing health industries, emphasizing personal health responsibilities, emphasizing the importance of prevention and a healthy lifestyle, and effectively controlling lifestyle behavioral factors that affect health. Under this policy, the prevention and control of chronic diseases have made positive progress and obvious results. However, the problem of obesity in China is still serious, and the prevalence of obesity is rapidly rising (1, 2). In a recent national survey, more than half of Chinese adults and one-fifth of children are overweight or obese (3), and another study investigated more than 1.5 million participants (median age 40 years; mean body mass index (BMI) 24.1 kg/m^2^; 52.8% male) and showed that 34.8% were overweight and 14.1% were obese according to China’s body mass index classification which indicates over 28 kg/m^2^ is obesity, over 24 kg/m^2^ regarded as overweight, and less than 18.5 kg/m^2^ classify underweight (4). Although there are regional and demographic differences, the gap in overall obesity rates between urban and rural areas is narrowing (3), which severely impacts the health status of individuals and increases their financial burden. At the same time, a series of therapeutics has been developed based on the causes and mechanisms of obesity, such as medical nutritional therapy, obesity management, and bariatric surgery (5). These implemented independent preventions and actions are still insufficient to effectively control the occurrence of obesity due to multiple factors (6), which is because the conditions that lead to obesity do not exist independently of each other. The complex interactions among biological, behavioral, social, and environmental factors impact the regulation of energy balance and fat storage (7).

The three-layered framework for studying obesity in China indicates that the growth of obesity in China is driven by unhealthy diets and physical inactivity, which can be magnified or modified at the individual level by genetic susceptibility, psychosocial factors, obesogens, and adverse early life exposures, among other concurrent potential risk factors (3). In addition, the WHO argues that insufficient physical activity is the 4th leading risk factor for mortality, having a 20–30% increased risk of all-cause mortality (8). Therefore, we explored the effects of interactions among risk factors on obesity at the individual level through physical activity and lifestyle habits to provide evidence for preventing, identifying, and managing obesity.

The results of previous studies suggest that physical activity and lifestyle have a significant influence on an individual’s obesity, regardless of diet. When living a bad lifestyle like poor sleeping which can lead to insufficient sleep, the secretion of adipokines such as leptin, lipocalin and endolysin, the secretion rhythm and the dynamic balance between them is disturbed (9). The metabolic changes of adipokines are also affected by PAHs derived from the combustion of cigarettes in the environment of secondhand smoke (10), as well as impacted by an accumulation of sedentary behaviors, which is accompanied by an increase in the duration of sedentary time leading to an accumulation in energy intake increases, further contributing to energy imbalance (11). Whereas increased physical activity improves energy expenditure and adjusts energy balance, at sufficient exercise intensity, exercise duration and exercise frequency have an impact on metabolism, which is positively correlated with a decrease in BMI (12). But there are differences in energy metabolism (13) and lipid metabolism levels (14) between exercise behaviors chosen to be performed at different times of the day. Moreover, education, as an upstream factor in health, sets the stage for an individual’s later health outcomes (15), that higher educational status has a lower correlation with obesity (16), and individuals with higher levels of education are better able to understand health knowledge (17).

**Figure 1:**
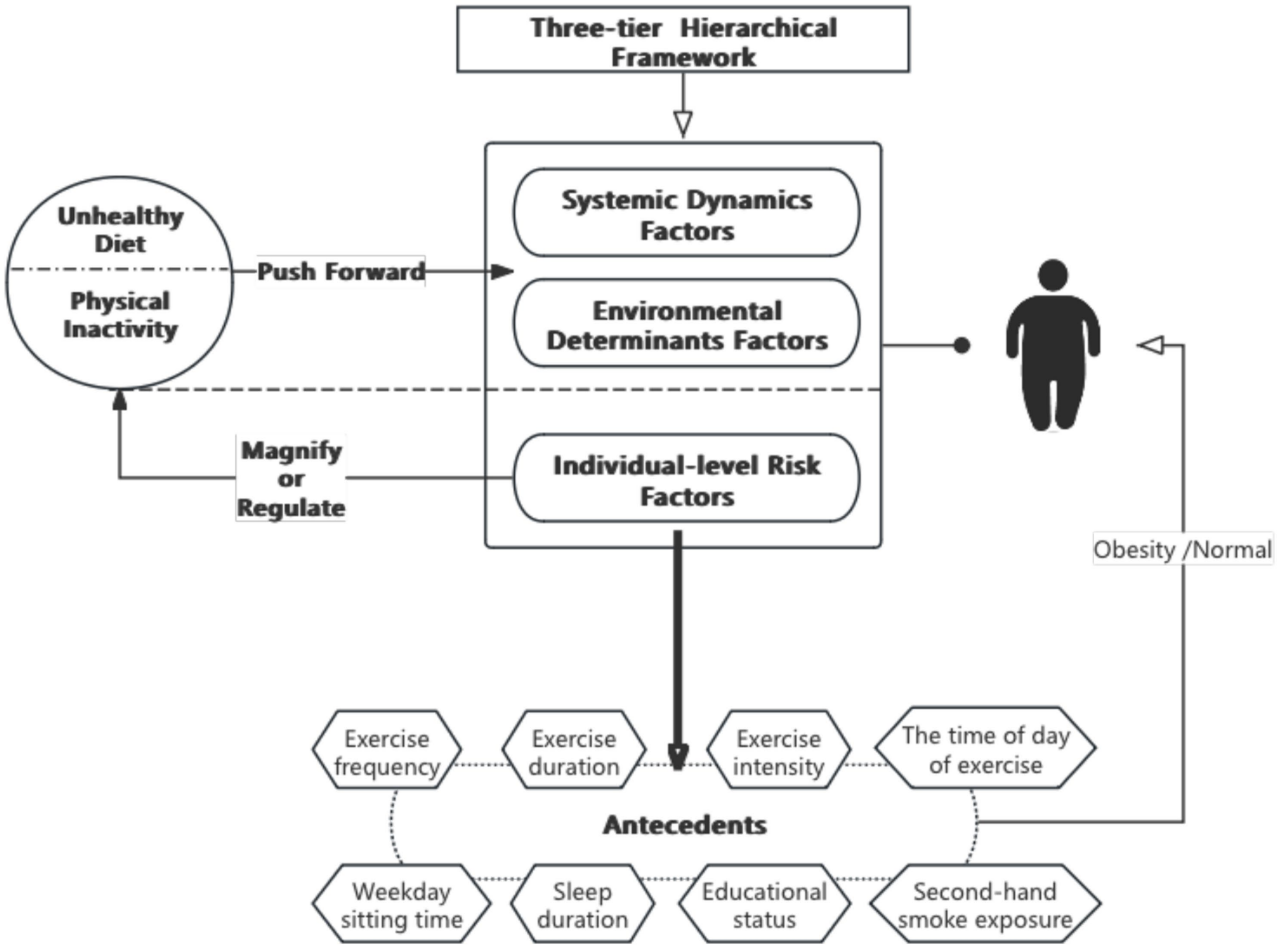
Logistic diagram of antecedent conditions.

Overall, there is an independent correlation between obesity, sleep duration, second-hand smoking exposure, weekday sedentary time, exercise duration, exercise frequency, time of day of exercise, exercise intensity, and educational status (18). In addition, based on the three-layered framework, with the physical activity and lifestyle of the individual risk factors as a base and BMI as the criteria for obesity to investigate whether the synergy effect between the eight independent factors that influence obesity can work on the BMI (Figure 1). In this context, we analyze conditions to find which combinations as the potential configurational path to influence the prevalence of obesity. And raises the question: Will obesity occur when the risk factors (insufficient sleep, second-hand smoke exposure, longer weekday sedentary time) and protective factors (exercise intensity, exercise duration, exercise frequency, exercise duration, educational status) are both present?

## 2 Method and data

### 2.1 Research methods

The configuration of physical activity and lifestyle that influence obesity is a complex and multiple concurrency process are influenced by multiple factors. Statistical analysis including correlational analysis and regression analysis is a variables-oriented method, it can quantify the net effects of individual variables and causal relationships. However, their capacity to deal with more complex theoretical issues is limited (19). So, based on the statistical analysis result combining the fsQCA to refine the potential causal relationships between explanatory variables and outcome variables.

The fuzzy-set qualitative comparative analysis (fsQCA) can deal with complex causal problems compared to traditional correlation. As a case-oriented method, fsQCA aims to analyze the data in comparative case studies, which is one type of Qualitative Comparative Analysis (QCA). Compared with other types of QCA, like crisp set QCA and multi-value QCA, fsQCA can analyze variables that are not binary by offering a more realistic approach which results in variables getting all the values within the range of 0–1 and computing degrees in which a case belongs to a set. And this handles causal complexity with fine-grained level data or identifies more solutions. So, it can precisely place cases relative to one another as well as interpret relevant and irrelevant variations (20). In addition, fsQCA identifies different configurations of sufficient and/or necessary conditions to focus on the complex and asymmetric relations between the outcome of interest and its antecedents (21). Also, it is suitable for different types of data such as Likert-scale, clickstreams, and multimodal data, and is available for different sample sizes, ranging from tiny sample sizes (<50 cases) to substantial sample sizes (thousands of cases) (20). Therefore, based on these rules, the fsQCA method can be applied to our study which is large numbers of cases, nearly thousands, including Likert-scale. And following steps will be implemented. (1) Sorting influenced conditions that are selected based on literature review and physical collection of data. (2) Using the correlation analysis to further validate the relationship between variables, and regression analysis to verify the quantitative relationship between variables. (3) Using empirical knowledge and related, and the results from statistical analysis to calibrate variables. (4) Based on the result of calibration to conduct necessity analysis knowing whether the key condition causes obesity. (5) Using configurations analysis to find out the necessary and sufficient conditions.

### 2.2 Data collection

The sample selected for the study came from the Healthcare Center at the Wuhan First People’s Hospital, located in Wuhan Hubei China. The Center provides various body examinations and health knowledge lectures, personalized recommendations on diet, lifestyle habits, disease prevention and treatment, personalized nutrition advice, and traditional Chinese Medicine Health Preservation services. Because the conditional variable data cannot be collected through instruments or laboratories, a questionnaire survey is used to collect the data. We used survey questionnaires to assess the condition of the respondents. The Physical Activity Readiness Questionnaire (PAR-Q) is a tool for pre-participation screening and risk stratification. The Physical Activity Survey Questionnaire investigates the fitness of respondents, and the Lifestyle and Chronic Disease Survey Questionnaire includes questions about items in your daily life related to health, such as sitting duration, second-hand smoking exposure, physical activities, chronic diseases, etc. We also analyzed the reliability of the questionnaire, and the result shows Cronbach’s alpha of the Lifestyle and Chronic Disease Survey Questionnaire is 0.98. The other two survey questionnaires use the non-Liker scale, which cannot use Cronbach’s alpha to analyze the reliability. To get the data accurate, all questionnaires were delivered face-to-face, and supervisors and healthcare center management told the participants how to fill the questions (understanding the questions) before collaboration. Also, Respondents were assured that there were no right or wrong answers and were encouraged to answer the questions as honestly as possible. Data collection took place between January 1, 2021, and January 31, 2022, after the lifting of the lockdown in Wuhan due to the epidemic.

**Figure 2:**
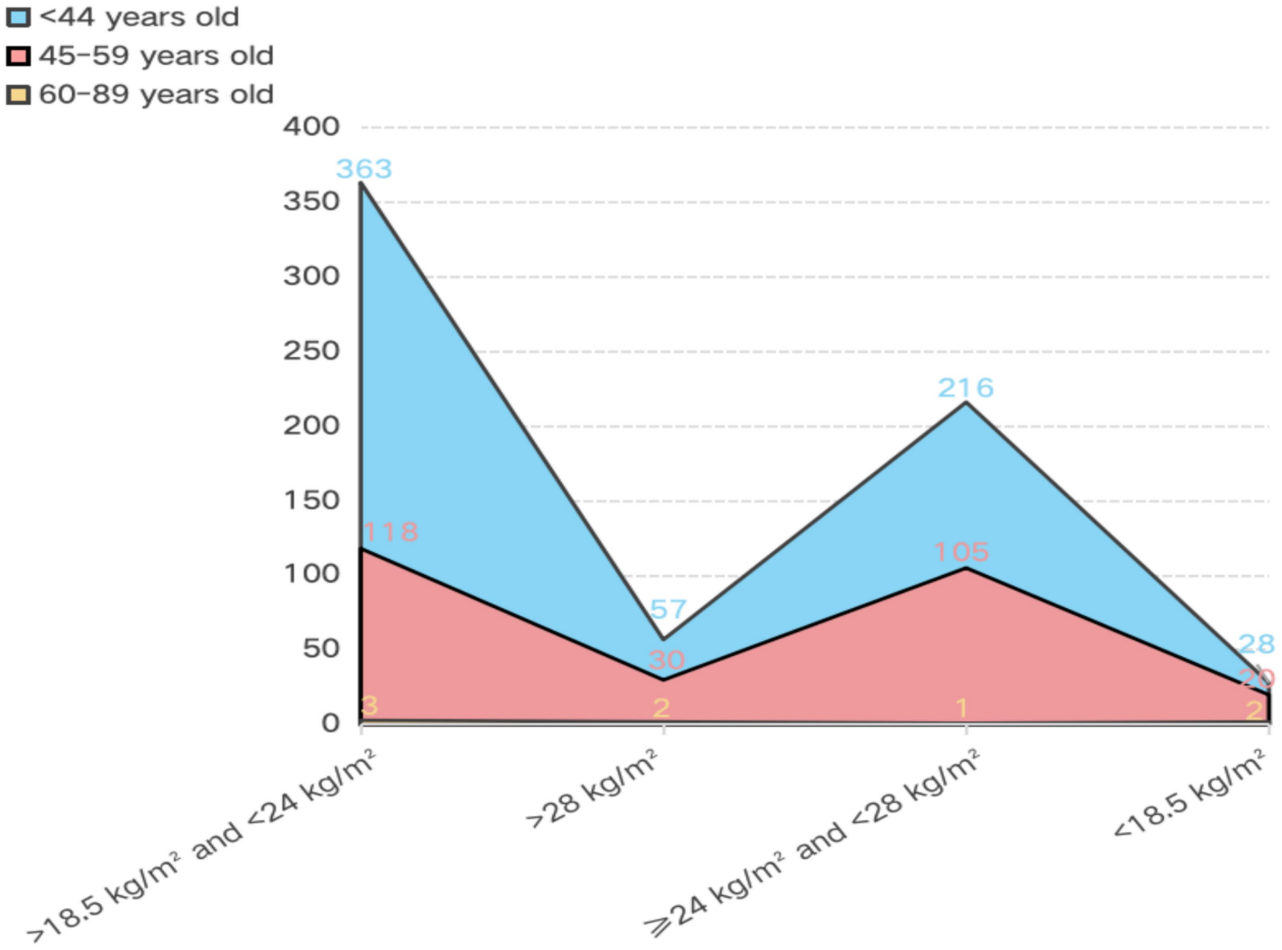
Distribution of body mass index by age. Explanation: The < 18.5, >18.5 and < 24, >28, ≧25 and < 28, are all the BMI indicators, the unite is kg/m^2^. Numbers on the vertical axis represent the number of cases.

Our study’s main goal is to find the combinatorial conditional antecedents of obesity in physical activity and lifestyle habits. Therefore, the questionnaire data was selected based on the antecedents in the literature review (Figure 1). There were 8 conditions and an outcome variable was involved (exercise frequency, exercise duration, exercise intensity, the time of day of exercise, weekday sitting time, second-hand smoke exposure, sleep duration, educational status, and body mass index). Table 1 lists the details of the antecedents including the categorical and continuous variables. 945 valid data were obtained by systematically excluding those who did not meet the requirements and those with missing answers. Of the 945 respondents, 52.8% were males and 47.2% were females, the oldest was 62 years old and the youngest was 20 years old, the average is 37.83 years old (Table 2). Figure 2 shows that BMI > 28 kg/m^2^ individuals is 89 (9.4%), BMI ≥ 24 kg/m^2^ and < 28 kg/m^2^ individuals is 322 (34%), BMI > 18.5 kg/m^2^ and < 24 kg/m^2^ individuals is 484 (51.2%), BMI < 18.5 kg/m^2^ individuals is 50 (5.2%). Among the obese group, their ages ranged from 22 to 62 years old (mean age, 37.83 years old), and over this age are 50 cases, and those younger than 44 years old have a higher risk of being obese when compared to those middle-aged at 45–59 years old.

**Table 1:** The description of variables.

Variables		Variable type	Indicators
Conditional variables	Exercise frequency	Categorical variables	1. Less than 1 time per month on average 2. More than 1 time per month on average, but less than 1 time per week 3. Average of 1 per week 4. Average of 2 times per week 5. Average of 3 times per week 6. Average of 4 times per week 7. More than 5 times per week on average
	Exercise duration		1. Less than 30 min 2. 30–60 min 3. 60 min and above
	Exercise intensity		1. Breathing and heart rate are not much different than when not working out 2. Rapid breathing and heart rate, slight sweating 3. Shortness of breath, markedly increased heart rate, heavy sweating
	The time of day of exercise		1. In the morning 2. In the afternoon 3. In the evening
	Second-hand smoke exposure		1. Everyday 2. An average of 4–6 days per week 3. An average of 1 to 3 days per week 4. None 5. Do not know/cannot remember
	Educational status		1. Never attended school 2. Literacy course 3. Primary school 4. Secondary school 5. High school or junior college 6. University (including junior college) 7. Postgraduate and above
	Sleep duration	Continuous variables	In Minutes (based on survey)
	Weekday sitting time		In Minutes (based on survey)
Outcome variable	BMI		BMI>28, Obesity BMI<24, Healthy Weigh BMI<18.5, Underweight

**Table 2:** Basic characteristics of the research sample.

Variables	Categories	Sample size	Percentage (%)	Mean ± SD
Age	<44	664	70.3%	37.83 ± 10.188
	45–59	273	28.9%	
	≥60	8	0.8%	
Sex	Male	499	52.8%	/
	Female	446	47.2%	
Educational status	Never attended school	7	0.7%	/
	Literacy course	4	0.4%	
	Primary school	29	3.1%	
	Secondary school	68	7.2%	
	High school or junior college	107	11.3%	
	University (including junior college)	629	66.6%	
	Postgraduate and above	101	10.7%	
BMI	<18.5 kg/m^2^	53	5.6%	23.61 ± 0.110
	18.6–24 kg/m^2^	495	52.4%	
	25–28 kg/m^2^	311	32.9%	
	>28 kg/m^2^	86	9.1%	

### 2.3 Data analysis

#### 2.3.1 Statistical analysis

**Figure 3:**
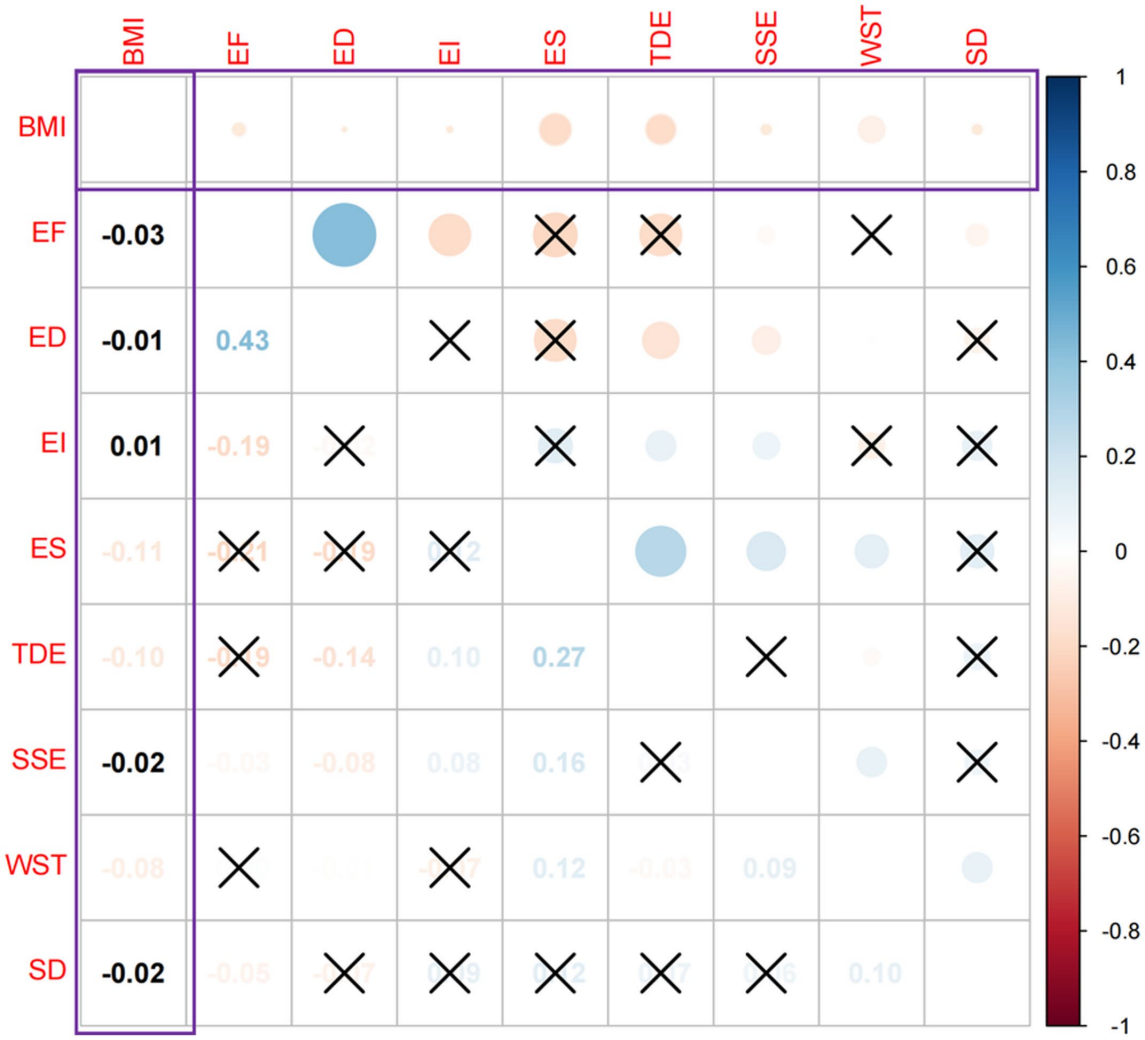
Relationship between BMI and influences. Explanation: × represents *p* > 0.05, in which no significant relationship between variables. The size of the bubble indicates the strength of the correlation between the two variables. The lighter the color, the lower the correlation (because some variables’ colors are so light, they can not be read, so turn it to black).

Before using fsQCA, we first test the relationships between BMI and all influencing factors, which is critical to better understanding whether a factor is important for supporting a high prevalence of obesity (Figure 3). There was a negative correlation between educational status and BMI (*r* = −0.11, *p* < 0.05), weekday sitting time and BMI (*r* = −0.08, *p* < 0.05), the time of the day of exercise and BMI (*r* = −0.10, *p* < 0.05), sleep duration and BMI (*r* = −0.02, *p* < 0.05), exposure to secondhand smoke and BMI (*r* = −0.02, *p* < 0.05), frequency of exercise and BMI were negatively correlated (*r* = −0.03, *p* < 0.05), duration of exercise and BMI were negatively correlated (*r* = −0.01, *p* < 0.05), and exercise intensity and BMI were positively correlated (*r* = 0.01, *p* < 0.05). The absolute values of the correlation coefficients in the negative correlations, from strongest to weakest, were educational status −0.11 > the time of the day of excise −0.10 > weekday sitting time −0.08 > frequency of exercise −0.03 > exposure to secondhand smoke −0.02 > sleep duration −0.02 > exercise duration −0.01. These findings are consistent with previous literature, confirming that both the practical experience and the statistical results of the data indicate that BMI is related to the above variables.

**Figure 4:**
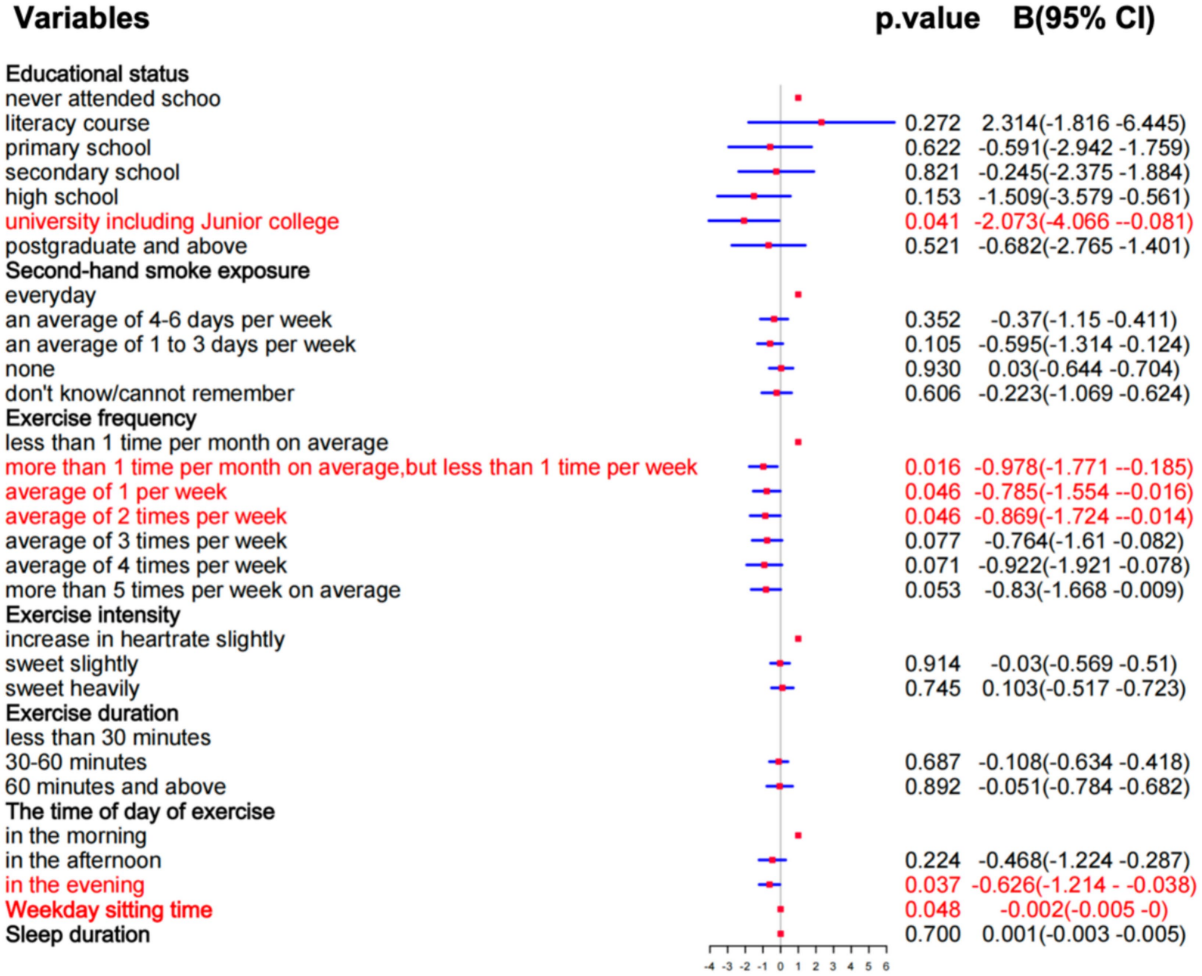
The outcome of regression analysis.

Variables affecting BMI were regressed based on the results of the correlation analysis to explore which factors had a significant impact on the results. From Figure 4, we know that all variables meet the standard of VIF, but only university (including junior college) (*p* = 0.041), more than 1 time per month on average, but less than 1 time per week (*p* = 0.016), average of 1 per week (*p* = 0.046), average of 2 times per week (*p* = 0.046), in the evening (*p* = 0.037), and weekly sitting time (*p* = 0.048) have a significant effect on BMI. Also, these factors have a negative correlation with BMI. The results show that these 6 factors affect obesity, but whether other independent variables will trigger the same outcome. Thus, based on the outcome of regression analysis we take further steps to find similar configurations, then provide a more in-depth examination of these cases.

#### 2.3.2 Variables calibration

Before analyzing the data by fsQCA, we must convert data from ordinal scales into degrees of membership in the target set. Each case is calibrated by assigning a value ranging from 0 to 1, which shows if and how much a case belongs to a specific set, it represents as full-set membership, intermediate-set membership, and full-set non-membership. After converting categorical variables and continuous variables into fuzzy sets, we calibrate the variables with three thresholds (full-set membership, intermediate-set membership, and full-set non-membership) for direct calibration and choose the values 95% (0.95), 50% (0.50), and 5% (0.05) as the breakpoints. If the data do not have a normal distribution but instead are skewed, then 80% (0.80), 50% (0.50), and 20% (0.20) can be set as the thresholds for full-set membership, intermediate-set membership, and full-set non-membership, respectively (20).

Antecedents, weekday sitting time and sleep duration, have a normal distribution, so the values 0.95, 0.50, and 0.05 as the breakpoints. And, some cases are exactly on 0.5 which makes it difficult to analyze the conditions that are set exactly on 0.5. To overcome this, we add a constant of 0.001 to the causal conditions to avoid the allocation of the 0.5 anchor (22). Likert scales have unique ways to calibrate, previous studies suggest that the values of 6, 4.5, and 2 can be used as thresholds for seven-point Likert scales and 4, 3.5, and 2 for a five-point Likert scale (20). So, conditions “educational status” and “exercise frequency” use 6, 4.5, and 2 as thresholds, and “second-hand smoke exposure” uses 4, 3.5, and 2. The conditions “exercise duration,” “exercise intensity,” and “the time of day of exercise” were measured using a set of 3 questions scales, the answers to each question were scored on a scale ranging from 1 to 3, thus, calibrating these three conditions with 1, 2, and 3. Regarding the outcome, BMI, based on China’s standards that obesity is BMI greater than or equal to 28 kg/m^2^, overweight is BMI greater than or equal to 24 kg/m^2^, underweight is BMI lower than 18.5 kg/m^2^ (3), following this approach, setting the 28, 24, 18.5 as the breakpoints (Table 3).

**Table 3:** Calibration anchor of conditions and outcome.

Feature	Explanation	Threshold value		
		Full-set membership	Intermediate-set membership	Full-set non-membership
C:EF	Exercise frequency	6	4.5	2
C:ED	Exercise duration	3	2	1
C:EI	Exercise intensity	3	2	1
C:TDE	The time of day of exercise	3	2	1
C:SSE	Second-hand smoke exposure	2	3.5	4
C:ES	Educational status	6	4.5	2
C:SD	Sleep duration	510.001	480.001	360.001
C:WST	Weekday sitting time	420.001	300.001	120.001
O:BMI	Body mass index	28	24	18.5

C, condition; O, Outcome; EF, Exercise frequency; ED, Exercise duration; EI, Exercise intensity; TDE, The time of day of exercise; SSE, Second-hand smoke exposure; ES, Educational status; SD, Sleep duration; WST, Weekday sitting time; BMI, Body mass index. The same anchors were used for analyzing the configurational path of non-BMI.

#### 2.3.3 Necessity analysis: the key cause of obesity

However, the information obtained from statistical significance was limited, so the statistical results were incorporated in conjunction with the fsQCA analysis to obtain more information. The main causes of obesity generation were extracted by analyzing the necessity of the antecedents, and the necessity outcome refers to its consistency. The consistency index indicates the proportion of samples that pass through this configurational path and achieve the outcome, the coverage index is the proportion of the number of samples passing through this configurational path to the total number of samples (21). Using 0.9 as the basic line, when the consistency of a single condition is achieved at 0.9 and has sufficient coverage at 0.5, we defined this condition as a necessary condition, meaning this condition always appears when the result exists. In other words, the outcome cannot be produced without this condition. The main purpose is to assess the subset relationship between the set of results and the set of individual conditions (23). Table 4 shows that when the outcome is “BMI” or “~BMI,” the consistency of “Educational Status” is 0.9, and coverage is 0.5 nearly, which means this antecedent condition strongly explains the results and can independently demonstrate the outcome. The other conditions do not achieve 0.9 and 0.5, which shows that the explanatory power of each single condition to the outcome, BMI, is weak.

**Table 4:** Analysis of necessary conditions for BMI.

Conditions	BMI		~BMI	
	Consistency	Coverage	Consistency	Coverage
EF	0.52172	0.57079	0.51378	0.62449
ED	0.59573	0.63440	0.58438	0.69141
EI	0.64274	0.61126	0.62836	0.66392
TDE	0.75386	0.50660	0.80179	0.59862
SSE	0.67601	0.61266	0.65214	0.65664
ES	0.89775	0.49999	0.94830	0.58677
SD	0.55044	0.68144	0.53371	0.73408
WST	0.63496	0.60747	0.643631	0.68411

EF, Exercise frequency; ED, Exercise duration; EI, Exercise intensity; TDE, The time of day of exercise; SSE, Second-hand smoke exposure; ES, Educational status; SD, Sleep duration; WST, Weekday sitting time; BMI, Body mass index (obesity); ~BMI, (non-obesity).

#### 2.3.4 Configurations analyzed: obesity generation configurational path

Based on empirical knowledge from necessity analysis, Educational Status as the “necessary condition” of the outcome, exercise frequency, the day of exercise, and weekly sitting time also affect the result from statistical analysis, thus setting these four independent variables being “present” in the configurations. By default, Others choose “present or absent,” assuming these conditions’ present or absent all may cause obesity. After that, the frequency threshold is set to 5 in truth tables, which means every configuration has 5 cases to support at least (24), and the consistency threshold is set to 0.8 in the truth table’s outcome. Firstly, because the PRI consistency’ stands for “Proportional Reduction in Inconsistency,” and is used to avoid simultaneous subset relations of configurations in both the outcome and absence of the result. So, we select truth table rows following this theory, when configurations with PRI scores below 0.5 indicate significant inconsistency (20). Therefore, the truth table rows of outcomes with PRI values less than 0.5 are manually modified to 0. Then the standardized analysis is performed to obtain three solutions: complex solution, parsimonious solution, and intermediate solution.

Select parsimonious solutions and intermediate solutions to identify the core conditions that appear in both solutions, which cannot be left out from any solution, and have a significant effect on the outcome. If the conditions only appear in the intermediate solutions, that is peripheral conditions meaning an auxiliary effect on the result (20). From the analysis, we concluded that “~ TDE” and “WST* ~ SD*SSE*EF*EI” are the core conditions (Table 5), and peripheral conditions in Table 6.

**Table 5:** Parsimonious solution for BMI.

Model: BIM = ES, WST, SD, SSE, EF, ED, EI, TDE			
Frequency cutoff: 5			
Consistency cutoff: 0.807547			
	Raw coverage	Unique coverage	Consistency
~TDE	0.402729	0.40279	0.646507
WST* ~ SD*SSE*EF*EI	0.195341	0.06324	0.803012
Solution coverage: 0.465978			
Solution consistency: 0.647619			

Explanation: “~” indicates the condition at a lower level, “*” means AND in the conditions set.

ES, Educational status; WST, Weekday sitting time; SD, Sleep duration; SSE, Second-hand smoke exposure; EF, Exercise frequency; ED, Exercise duration; EI, Exercise intensity; TDE, The time of day of exercise; ~TDE, exercising in the morning.

**Table 6:** Intermediate solution for BMI.

Model: BIM = ES, WST, SD, SSE, EF, ED, EI, TDE			
Frequency cutoff: 5			
Consistency cutoff: 0.807547			
Assumptions: EF\TDE\ES\WST (present)			
	Raw coverage	Unique coverage	Consistency
~SD*SSE*EF*ED* ~ TDE	0.181805	0.052918	0.824076
ES* ~ SD*SSE*ED*EI* ~ TDE	0.158707	0.006487	0.833604
ES*WST*SD*ED*EI* ~ TDE	0.153243	0.003600	0.867101
ES*WST*EF*ED*EI* ~ TDE	0.144407	0.005803	0.882367
ES*WST* ~ SD*SSE*EF*ED*EI	0.167406	0.048437	0.810588
ES*WST* ~ SD* ~ SSE* ~ EF* ~ ED*EI* ~ TDE	0.126351	0.0213448	0.881632
Solution coverage: 0.312787			
Solution consistency: 0.766165			

## 3 Results

### 3.1 Analysis of the outcome variable: obesity

The pathway that causes the result is shown in Table 7, in which six configuration paths produce BMI (obesity). The consistency indexes of the six configurations are 0.82407, 0.83360, 0.86710, 0.88236, 0.81058, and 0.88163, respectively, all of which are higher than 0.8, which shows that all configurations are sufficient conditions for BMI. The overall solution coverage is 0.31278, indicating that about 30% of results are explained by all combinations of conditions (six configurations).

**Table 7:** The configurational path of the BMI.

Conditions	Pathway A	Pathway B	Pathway C	Pathway D	Pathway E	Pathway F
ES		●	●	●	●	●
WST			●	●	●	●
SD	⊗	⊗	●		⊗	⊗
SSE	●	●			●	⊗
EF	●			●	●	⊗
ED	●	●	●	●	●	⊗
EI		●	●	●	●	●
TDE	⊗	⊗	⊗	⊗		⊗
Consistency	0.82407	0.83360	0.86710	0.88236	0.81058	0.88163
Raw coverage	0.18180	0.15870	0.15324	0.14440	0.16740	0.12635
Unique coverage	0.05291	0.00648	0.00360	0.00580	0.04843	0.02134
Solution consistency	0.76616					
Solution coverage	0.31278					

Black circles (●) indicate the presence of a condition, and circles (⊗) indicate its absence. Large circle: core condition, Small circle: peripheral condition, blank space: “do not care” condition.

ES, Educational status; WST, Weekday sitting time; SD, Sleep duration; SSE, Second-hand smoke exposure; EF, Exercise frequency; ED, Exercise duration; EI, Exercise intensity; TDE, The time of day of exercise.

Like what is expressed in Table 7, the results show that six configuration paths resulted in BIM, and these paths affect the development of obesity. The core conditions for configuration pathways B, C, D, and F are the same: higher educational status (ES ●) and morning exercise (TDE ⊗). The core conditions of pathway E are higher educational status (ES ●), Weekday sitting time of about 7 h (WST ●), Sleep duration of about 6 h (SD ⊗), Second-hand smoke exposure on average of 4–6 days per week (SSE ●), exercise frequency on average of 4 times per week (EF ●), exercise intensity on shortness of breath, markedly increased heart rate, heavy sweating (EI ●). Table 8 shows every pathway that can explain how many cases of BMI there are.

**Table 8:** The details of configurational pathways of the BMI.

Configuration pathway	BMI < 18.5 kg/m^2^	BMI < 24 kg/m^2^	24 kg/m^2^ ≤ BMI < 28 kg/m^2^	BMI > 28 kg/m^2^
Pathway A	5 (11.9)	10 (23.80)	21 (50)	6 (14.3)
Sex	4/M,1/F	3/M,7/F	2/M,19/F	2/M,4/F
Age (average)	47.25	46.8	48.7	47.3
Pathway B	3 (17.6)	8 (47.1)	4 (23.5)	2 (11.8)
Sex	2/M,1/F	5/M,3/F	2/M,2/F	2 M
Age (average)	53	39.5	44.25	47
Pathway C	1 (4.5)	5 (22.7)	9 (40.9)	7 (31.8)
Sex	1/M	2/M,3/F	5/M,4/F	5/M,2/F
Age (average)	33	36.4	39.3	44
Pathway D	1 (4.8)	7 (33.3)	7 (33.3)	6 (28.6)
Sex	1/F	4/M,3/F	5/M,2/F	4/M,2/F
Age (average)	45	36.9	44	43
Pathway E	3 (16.7)	9 (50)	5 (27.8)	1 (5.6)
Sex	2/M,1/F	5/M,4/F	3/M,2/F	1/M
Age (average)	54	40.5	46	46
Pathway F	/	/	1 (100)	/
Sex	/	/	1/F	/
Age (average)	/	/	32	/

The pathway A, its peripheral conditions are sleeping around 6 h, exposure to second-hand smoke for 4–6 days per week, exercising for more than 60 min, and maintaining a frequency of 4 times a week. This suggests that, regardless of the exercise duration and frequency, if someone has all of these habits together, the prevalence of obesity is higher than others. The pathway B, its peripheral conditions are sleeping around 6 h a day, exposure to second-hand smoke for 4–6 days per week on average, exercising for more than 60 min, and keeping in a relatively high intensity with increased heart rate and heavy sweating. This combination of habits can explain why someone may be at risk of being obese. The configuration pathway C peripheral conditions are sitting around 7 h during the working days, sleeping around 8.5 h per day on average, exercising for more than 60 min, and keeping in a relatively high intensity with increased heart rate and heavy sweating. The fourth combination of causes (D), its peripheral conditions are sitting around 7 h during the working days, exercising for more than 60 min, and keeping in a relatively high intensity with increased heart rate and heavy sweating. The fifth (E) affected pathway’s peripheral conditions are sitting around 7 h during the working days, sleeping around 6 h per day, exposing to second-hand smoke for 4–6 days per week on average, maintaining a frequency of 4 times a week and lasting 60 min or above per time, and keeping in a relatively high intensity with increased heart rate and heavy sweating. The last pathway (F), its peripheral conditions are sitting around 7 h during the working days on each day, sleeping around 6 h a day, not being exposed to a second-hand smoking environment, exercising more than 1time per month, but less than 1time per week, and keeping in a relatively high intensity with increased heart rate and heavy sweating last less than 30 min per time. The distribution of sex and age of each configuration pathway are detailed in Table 8.

## 4 Discussion

From the results, we know that different combinations of lifestyles and physical activity affect the prevalence of obesity, even though some individuals are actively exercising they are still at risk of obesity when living a variety of bad lifestyles. So, not all risk factors (insufficient sleep, second-hand smoke exposure, longer weekday sedentary time) and protective factors (exercise intensity, exercise duration, exercise frequency, exercise duration, educational status) are present that will cause obesity to occur. Of all of the factors, educational status as the necessary factor that influences the outcome most, namely, the data in research shows that a BMI over 28 kg/m^2^ educational status must be involved.

### 4.1 Geographic and environmental factors

According to both WHO and Chinese BMI classifications, the prevalence of overweight and obesity was higher in northern China than in southern, with the highest prevalence generally seen in Inner Mongolia, Shandong, and Hebei. A correlation with Gross Domestic Product (GDP) per capita was explained this, with a greater prevalence of overweight and obesity among participants from lower GDP per capita regions (4). From the GDP data of 31 provinces in 2023, Hubei Province ranked seventh, with the annual GDP reaching 5,580.363 billion yuan (25), as the provincial capital, Wuhan’s gross domestic product (GDP) was 200.165 billion yuan in 2022 (26), around 3.6% of the province’s GDP (17 cities). We all know that higher education has a significant impact on GDP growth (27), as government report, in 2022, Wuhan had 83 institutions of higher education (28), among the city’s resident population, there are 417.46 million people with university education (college and above), 33.87% of the city’s resident population (The above levels of education include graduates, incompletes, and current students of all types of schools) (29).

Furthermore, environmental factors, such as walkable neighborhood designers, access to parks, availability of public transit, and quality of pedestrian and bicycling infrastructure, affect obesity through physical activity and play roles in physical activity (30), and higher walkable areas are associated with lower BMI or overweight prevalence (31). In 2023, the Wuhan government constructed the physical-supportive environment “12-min fitness circle,” to facilitate and provide convenience to citizens participating in physical activities. This infrastructure is around 12.3 million square meters, including basketball halls, badminton halls, tennis halls, soccer fields, constant-temperature swimming pools, tide play sports halls, learning and training centers, sports rehabilitation centers, lodging service centers, health and fitness testing centers, extreme sports parks, children’s parent-children’s parks, and other sports and leisure facilities. It addresses the problem of insufficient sports venues and facilities in the area, to meet the demand for sports and fitness of nearly 500,000 residents in dozens of surrounding neighborhoods. Moreover, Wuhan now has a total of more than 40,000 sports venues and facilities, 52 sports parks of various types, the city’s streets (townships), community (village) fitness facilities system completed, the city’s 24 public stadiums at both levels of the free and low-fee opening of the full implementation (32). In addition, the government plans to complete the investment of 14.028 billion yuan in urban construction of landscaping and greening, 100 new and reconstructed various types of parks, 100 kilometers of greenways, and increasing green open space (33).

Even though Wuhan with such abundant, unique regional resources, we are still exposed to obesity from the data analysis. For one reason, the “12-min fitness circle” was constructed later than the data collection. Another, this policy does not cover the whole city so far. Furthermore, the educational level in this study has to be analyzed in further depth, due to it plays a significant role in the configurational path of obesity.

### 4.2 Educational status as a key factor in obesity

Among all the factors in this study, the results reveal a strong correlation between obesity and educational level, the education-obesity nexus. According to the configurational paths that cause the development of obesity, outcomes show the changes in educational status as a core condition are theorized to have a significant effect on obesity, implying that educational status substantially impacts the development of obesity. Similar results were obtained in a previous survey of the United States in 2022, showing that the prevalence of adult obesity decreased as education level increased (34).

Firstly, Individuals who have an educational level below or equivalent to primary school (never attended school, or literacy course) might have a lower level of health awareness among the more educated, and a practice that is largely dependent on the available knowledge. The study shows an improvement in attitude regarding obesity after receiving online education, Proving that changes in their knowledge have a positive effect and will influence their awareness of obesity (35). Also, education enables individuals to develop a broad range of skills and traits that predispose them toward improved health outcomes (36). In addition, the level of educational status is directly linked to the level of health literacy, affecting the ability of individuals to obtain, understand, and use basic health information and services to make health-related decisions and actions. In the knowledge-based information sources, along with the improvement of individual education level, the proportion of available information sources transformed into available sources has increased significantly, affecting the use of acquired information, it allows individuals to find better care to reduce the damage and have sound decision-making by using information more effectively (37).

Secondly, higher levels of education enable individuals to earn higher incomes, promote healthy lifestyles, and increase access to healthcare services (38). People with higher education and good health could easily convert their human capital into a multidimensional economic return by acquiring high-profile jobs (39), those with higher levels of education have higher coefficients and levels of work. In comparison, those with lower levels of education use more health capital to generate income, resulting in higher depreciation rates for health capital (17). Also, educational level may prevent individuals from certain physical environmental risk factors, such as air pollution, toxins, and lead, as well as decrease job stress (40). All in all, these factors may be the reason why educational status becomes the core condition in the occurrence of obesity in our study.

### 4.3 Multiple factor interactions as a main configurational path in obesity

The six configurational paths all include protective factors and risk factors, so we have to comprehensively understand the causes. A previous study showed sleeping less than 6 h per night has been linked to an increased likelihood of obesity (41), under presumed negative energy balance conditions in older and younger adults, combined circadian misalignment and insufficient sleep increased blood levels of ghrelin and decreased blood levels of leptin, which should promote energy intake (42). The nocturnal wakefulness as well as affects the level of Nicotinamide phosphoribosyltransferase (Nampt/visfatin/pre-B-cell colony-enhancing factor), the stronger the phase-shifting impact of nocturnal wakefulness on the regulation of Nampt levels, the more pronounced the impairing effect of sleep loss on glucose tolerance becomes (43). Also, Inefficient night-time sleep decreased levels of fibroblast growth factor 19, which is a protein increasing energy expenditure (42).

The causal relationship between sleep deprivation and obesity may be due to a common potential factor of exercise. However, exercise at different times of the day can have different effects on physical performance, for example, vigorous exercise before 10 p.m. may improve sleep quality. But, exercise that is too strenuous and too close to bedtime can cause a stress response (44), as taking physical activity too late may increase the rectal temperature during nighttime sleep, counteracting the positive effect of exercise in sleeping (45), the result caused by sleep deprivation or poor quality can disrupt the circadian rhythm, leading to metabolic disturbances that can lead to obesity (46). However, increased physical activity in proper time can attenuate the effect of the fat mass and obesity-associated (FTO) locus on obesity risk by 30–40% (47). The FTO regulates energy expenditure, when FTO deficiency it results in the upregulation of uncoupling protein 1 (UCP-1) in adipocytes, thus, enhancing mitochondrial uncoupling and energy expenditure in brown adipocytes (48). Also, the variants in the FTO locus directly interacts with the promoter of Irx3 and result in its deficiency, which can reduce weight and increase metabolic with the browning of white adipose tissue (47). One of the adipokines’ leptin acts in an autocrine/paracrine manner, regulating the white adipose tissues’ browning process. After physical activities, the adipokine leptin stimulates activity in the sympathetic nerve and together with insulin acts synergistically in different neuronal subsets of proopiomelanocortin (POMC) inducing browning of White Adipose Tissue (WAT) through decreased hypothalamic inflammation caused by exercise (49).

In addition, The WHO Guidelines on physical activity suggest adults aged 18–64 years including those with chronic conditions and those living with disability a strong recommendation may increase moderate-intensity aerobic physical activity to >300 min or do >150 min of vigorous-intensity aerobic physical activity, or an equivalent combination of moderate-intensity and vigorous-intensity activity throughout the week for additional benefits (50). Participants involved in our study aged between 20 and 62 years correspond with this recommendation, but their exercise intensity “Breathing and heart rate are not much different than when not working out” does not meet the standard of the moderate intensity and exercise duration recommended, even though the physical activity of light intensity provides health benefits, it might not achieve the weight loss targets to mitigate obesity risk through energy expenditure. Thus, they are still physical inactivity. Physical inactivity and sedentary behavior may increase the risk of obesity, especially prolonged and uninterrupted sedentary behavior patterns. However, there is a potential benefit of shortening sedentary behavior bouts and increasing sedentary behavior breaks. Because brief muscle contractions during a sedentary behavior break may improve blood flow and promote glucose uptake and homeostasis (51).

Besides energy expenditure through exercise, and decreasing or interrupting sedentary, lipolysis in adipose tissue should be considered. Benzo[a]pyrene(B[a]P) abundant in side-stream smoke [side-stream smoke contributes to 80% of secondhand smoke (52)], inhibited the *β*-adrenergic stimulation of lipolysis in adipose tissue in mice (53). The catecholamine-induced lipolysis in mouse adipocytes and isolated human adipocytes was inhibited by B[a]P, a polycyclic aromatic hydrocarbon (PAH) that is abundant in second-hand smoke exposure (53). Inhibition of lipolysis by B[a]P proceeds via direct inhibition of the early step of β-adrenergic receptor and ACTH receptor signaling to their respective G-coupled proteins (54). Beyond that, one at a lower education level has a bias in understanding the information they receive about exercise and health and a higher probability of misinterpreting health information (55).

In the process of BMI reduction, we should consider the multiple concurrent factors of physical activity and living habits, because we have to consider the “dose–response relationship” between these behaviors and obesity. Another research shows obesity management should take a holistic approach addressing multiple factors that include lifestyle modifications (56). However, the study sample in our research is relatively young (<44 years old: 70.3%) and well-educated (University including junior college: 66.6%), although this young age is consistent with the peak prevalence of obesity age in the Chinese population (35–54, 57). So, other research should be conducted in the future to certify the multiple-factor intervention’s effectiveness because the analysis is more theoretical.

## 5 Conclusion

This study primarily established the correlation between physical activity and lifestyle in obesity. Through the analysis of the case study, extracted and identified the necessary conditions and configurational paths that cause obesity. Combined with the data results and previous studies, the following conclusions were drawn: (1) The occurrence of obesity is closely related to the level of education. (2) In the development of obesity, the interaction of multiple influencing factors needs to be integrated and considered.

## Data Availability

The raw data supporting the conclusions of this article will be made available, upon reasonable request to the corresponding author LT tanglixu@126.com.
